# Development and validation of a questionnaire to determine medical orders non-adherence: a sequential exploratory mixed-method study

**DOI:** 10.1186/s12913-021-06147-3

**Published:** 2021-02-12

**Authors:** Vahid Yazdi-Feyzabadi, Nouzar Nakhaee, Mohammad Hossein Mehrolhassani, Soheila Naghavi, Enayatollah Homaie Rad

**Affiliations:** 1 Health Services Management Research Center, Institute for Futures Studies in Health, Kerman University of Medical Sciences, Kerman, Iran; 2 Department of Health Management, Policy and Economics, Faculty of Management and Medical Information Sciences, Kerman University of Medical Sciences, Kerman, Iran; 3 Neuroscience Research Center, Institute of Neuropharmacology, Kerman University of Medical Sciences, Kerman, Iran; 4 Medical Informatics Research Center, Institute for Futures Studies in Health, Kerman University of Medical Sciences, Kerman, Iran; 5 Social Determinants of Health Research Center, Institute for Futures Studies in Health, Kerman University of Medical Sciences, Kerman, Iran; 6 Social Determinants of Health Research Center, Guilan University of Medical Sciences, Rasht, Iran

**Keywords:** Non-adherence, Prescriptions, Ambulatory care facilities, Instrument development, Psychometric evaluation

## Abstract

**Background:**

Patients’ non-adherence with medical orders of physicians in outpatient clinics can lead to reduced clinical effectiveness, inadequate treatment, and increased medical care expenses. This study was conducted to develop and validate a questionnaire to determine the reasons for patients’ non-adherence with physicians’ medical orders.

**Methods:**

A sequential exploratory mixed-method study was conducted in two stages. The first stage comprised a qualitative stage to generate the primary items of the questionnaire. This stage provided findings of two sub-stages comprising a literature review and the findings of a qualitative conventional content analysis of 19 semi-structured interviews held with patients, physicians, and managers of the outpatient clinics in Kerman, an area located in southeastern Iran. The second stage comprised a quantitative study aiming evaluation of the instrument psychometric properties, including the face, content, construct, and reliability assessment of the questionnaire. Construct validity assessment was evaluated using exploratory factor analysis (EFA). The reliability assessment was done using assessing internal consistency (Cronbach’s alpha). To assess the construct validity of the questionnaire, four hundred and forty patients referred to outpatient clinics in Kerman were selected using stratified convenience sampling to fill out the questionnaire. The sample size was calculated using the Cochran formula. Qualitative and quantitative data were analyzed by MAXQDA 10 and Stata version 14, respectively.

**Results:**

The primary items contained 57 items, of which 42 met the minimum acceptable value of 0.78 for item-level content validity index (I-CVI = 1 for 24 items and I-CVI = 0.8 for 18 items). Item-level content validity ratio (I-CVR) was confirmed for 18 items with a minimum acceptable value of 0.99 for five experts. Finally, 18 items obtained the acceptable value for both I-CVI and I-CVR indicators and were confirmed. Using EFA, four factors (intrapersonal-psychological, intrapersonal-cognitive, provider-related, and socio-economic reasons) with 18 items and Cronbach’s alpha coefficient of 0.70, 0.66, 0.73, and 0.71, respectively, were identified and explained 51% of the variance. The reliability of the questionnaire (*r* = 0.70) was confirmed.

**Conclusion:**

The questionnaire with four dimensions is a valid and reliable instrument that can help determine the perceived reasons for non-adherence with medical orders in the outpatient services system.

**Supplementary Information:**

The online version contains supplementary material available at 10.1186/s12913-021-06147-3.

## Background

Problems related to patients’ non-adherence with medical orders are among the main concerns of the healthcare system. Suppose patients do not comply with therapeutic recommendations of physicians, despite the efforts of physicians. In that case, the desired therapeutic outcomes are not achieved, and it is the primary concern in the clinical field [1]. Hence there is a need to find an instrument to detect medical orders non-adherence.

Healthcare adherence is related to the individual’s ability to keep medical orders prescribed by physicians. It includes the timely presence in the predetermined treatment programs, the timely and proper use of medicines, follow-up of the disease for any later required referral, and adherence with health behaviors change. In healthcare non-adherence, patients do not comply with therapeutic recommendations consciously and intentionally [2].

World Health Organization (WHO) defines therapeutic adherence as “the extent to which a person’s behavior (in terms of taking medications, following diets, or executing lifestyle changes) coincides with medical or health advice” [3]. Otherwise, non-adherence occurs.

Non-adherence may lead to frustration for medical providers [4] and result in undesirable clinical outcomes, increased use of health services [5], increased rate of diseases and mortality [6], and waste of resources in healthcare systems [7].

Studies on various diseases have shown adverse outcomes of the non-adherence, such as the risk of death in HIV-infected patients [8], risk of mortality in patients suffering cancer [6], increase in the risk of diabetes [9], and medical costs for cancer, diabetes, and cardiovascular disease increase over twice [10]. Additionally, 10% of hospitalization in the elderly is due to medication non-adherence [10, 11]. Outpatient clinics are the first contact of patients and the community, and many hospital admissions are taking place through these clinics [12]. Non-adherence with therapeutic recommendations is one of the reasons for the disease’s progression and complications [13, 14], prolonged treatment period, and increased health care costs [15–17]. Effective interventions need to be developed to improve patients’ adherence and improve clinical outcomes.

There are various instruments to assess adherence and non-adherence in different diseases [18–21]. For example, Sidorkiewicz et al. developed an instrument to assess adherence for each drug taken by patients, usable in hospital and primary care settings [22]. Another study conducted in a developing country developed an approved instrument that measures medication adherence in patients with chronic diseases [23]. Another instrument for non-adherence was developed and validated, which was focused on intentional non-adherence [24]. However, existing instruments are disease and order specific, which does not include the most relevant of patients’ perceived reasons for non-adherence with medical orders generally in outpatient clinics, irrespective of disease, and prescription types. In this regard, to develop a valid and reliable instrument covering most relevant the perceived reasons for medical orders non-adherence can help the policy makers understand and identify the reasons and consequently adopt tailored policies to improve adherence [25, 26]. Thus, the present study aimed to develop a questionnaire and evaluate its psychometric properties to determine the perceived reasons for patients’ non-adherence with physicians’ medical orders in the outpatient clinics.

## Methods

The present study was a mixed-method study with a sequential exploratory design. This study was conducted in two stages. The first stage aimed to conduct a qualitative study to generate a pool of the questionnaire’s primary items. This stage focused on findings of two sub-stages comprising a literature review and a qualitative conventional content analysis findings. The second stage followed a quantitative study to evaluate the psychometric properties of the instrument.

We held nineteen semi-structured interviews with patients, physicians, and managers of the outpatient clinics in Kerman, an area located in southeastern Iran. The second stage comprised a quantitative study aiming evaluation of the instrument psychometric properties, including the face, content, construct validity, and reliability assessment of the questionnaire. Construct validity assessment was evaluated using exploratory factor analysis (EFA).

### Literature review and qualitative inquiry

Literature review and qualitative study were conducted to identify the reasons for non-adherence with medical orders and generally facilitate item generation [27]. We first conduct a literature review to identify the existing questionnaires and extract the reasons related to non-adherence with therapeutic orders prescribed by physicians from previous studies. Several valid scientific databases in Persian and English languages were aimed to retrieve the most relevant information about non-adherence with medical orders. The Persian databases of Scientific Information Database (SID), Iranian Magazines (Magiran), and Iranian Research Institute for Information Science and Technology (IranDoc) and English databases of Web Of Science (WoS), PubMed, Scopus, and google scholar were searched. The search strategy will include only terms relating to or describing non-adherence with medical orders. The most relevant keywords used were non-compliance, compliance, non-adherence, adherence, outpatient, ambulatory care, ambulatory care facilities, therapeutic recommendation, therapeutic orders, and medical orders. Definition of non-adherence and its dimensions and reasons were extracted, and many items were generated.

Furthermore, a qualitative study with a conventional content analysis approach was conducted to complete the reasons for non-adherence with medical orders in the setting of outpatient clinics. In this sub-stage, we conducted 19 semi-structured interviews with patients (*n* = 10), physicians (*n* = 5), and managers of the outpatient clinics (*n* = 4) in Kerman, an area located in southeastern Iran. Physicians, health care managers, and medical officials were selected using the purposeful sampling method, and patients were selected using convenience sampling. Qualitative data were analyzed using conventional content analysis by MAXQDA version 10. Qualitative data contribute to the enrichment and development of the concept and is a valuable resource for developing instruments [28]. More details of the results of the qualitative study were reported elsewhere [29]. The findings obtained from the literature review and the codes derived from the qualitative data analysis were mixed together. Eventually, a list of the most relevant reasons related to non-adherence with medical orders was identified. A first draft of the questionnaire was prepared, and included items were reviewed several times to ensure appropriate wording of the items, remove duplicates, and arrange their arrangement.

The questionnaire for non-adherence with therapeutic recommendations was scored based on a 5-point Likert scale (0 = very unimportant, 1 = unimportant, 2 = slightly important, 3 = important, 4 = very important). Patients at least 18 years old who experienced at least once non-adherence with medical orders during the past 4 weeks were considered the target group to fill out the questionnaire. The questionnaire was self-administered by patients. It consisted of 18 items, and the higher scores represented the higher importance of a given reason or factor. The questionnaires were completed anonymously and in a self-reported manner to overcome social desirability bias [30]. To minimize the end aversion bias, we tried to make questions clear for respondents.

### Evaluation of the psychometric properties of the instrument

At this stage, the instrument’s psychometric properties, including the face, content, construct validity, and reliability (internal consistency), were assessed.

### Face and content validity assessment

At this stage, the approval of experts for the content of the instrument was necessary. Therefore, after obtaining the experts’ approval for the instrument’s content and recording their comments, the instrument was drafted, the required changes in the instrument were made.

Content validity ratio (CVR) and content validity index (CVI) for each item and scale were calculated to determine the questionnaire’s content validity [26]. To determine the necessity and to include the most necessary items, the CVR, and to ensure the relevance, the CVI, was used [31]. The opinions of five experts with research backgrounds in questionnaire development and adherence and compliance in the health were surveyed. In the questionnaire given to the experts, it was explicitly explained which concept is measured by the questionnaire to give their opinion with enough knowledge about the subject.

The experts were asked to respond to 57 items based on scoring each item from 1 to 3 with a three-degree range of 1 = not necessary, 2 = useful but not essential, 3 = essential. Then, based on the experts’ responses, the CVR was determined using the following equation, and each item was scored and conformed with the Lawshe table for the number of experts involved.

$$ CVR=\left({N}_e-N/2\right)/\left(N/2\right) $$

Where *N*_*e*_ is the number of experts who rated the item as necessary, *N* is the total number of experts who rated the item. The acceptable value for the questionnaire’s validity is determined according to the Lawshe Table (1975) [32]. According to this table, in the case of involving five experts, items with a validity score higher than 0.99 were considered acceptable in this study.

The experts were asked to score 57 items based on a 4-point Likert scale (1 = completely irrelevant, 2 = partially relevant, 3 = greatly relevant, 4 = completely relevant) to determine the degree of relevance. The CVI was calculated by dividing the total number of experts that scored 3 or 4 by the total number of experts. The items with a CVI of 0.78 and higher were considered acceptable [33].

The face validity is an objective judgment about the construct of an instrument, which indicates the instrument’s relevance to the study’s aim, how to express phrases, the wording of questions, and understanding the researcher’s intended concept [34]. The experts were asked to give their opinions on the items’ content regarding the main question of the study and items’ wording (literary editing and fluency of terms and words) and, if necessary, express their suggested item. Also, for assessing the questionnaire’s comprehensibility by the target group, an interview was conducted with ten low-educated patients by one of the researchers to find the difficulty, the possibility of ambiguity, and inappropriate comprehension of the expressions.

### Construct and factorial validity

#### Sampling, setting and data gathering

The sample size was calculated using Cochran’s formula for descriptive cross-sectional studies to measure the rate and perceived reasons for the non-adherence with physicians’ medical orders. A previous study was not found in Iran to measure generally the types of non-adherence with medical orders. Thus, the value of p and q was considered 0.5 (the state in which it gives the maximum variance), and z and d (margin of error) were regarded to be 1.96 and 0.05, respectively. The primary sample was calculated to be three hundred and eighty-five samples. As some questionnaires might be incompletely filled, 15 % (15%) was added to the primary sample size. So, the final sample size was considered to be four hundred and forty samples.

The patients were selected using a two-stage stratified convenience sampling method. First, the outpatient clinics by ownership type, including clinics affiliated with social security, armed force, medical university, private and non for profit, were considered strata. The number of clinics in each stratum was selected proportionally to the total number of clinics. Furthermore, considering socio-economic variation, the clinics were randomly selected across the geographical areas to cover the most advantaged areas to the most disadvantaged ones. Second, one of the researchers referred to sampled clinics, and four hundred and forty patients were selected based on convenient sampling during 1 month (4 weeks), twenty-five patients from each clinic. The questionnaires were self-administered and personally distributed by the researcher after explaining the research’s purpose to respondents. The face-to-face interview was conducted if the patients were illiterate.

#### Construct validity

Construct validity is the degree to which the instrument is consistent with the theory. It is measured and could be established in different ways, such as examining the new instrument’s correlation with a well-validated questionnaire, distinguishing one group from the other based on some crucial variables [35], different forms of factor analysis, and other statistical evaluations. We had no well-established scale measuring non-adherence in the Persian language, irrespective of disease and medical order types. Therefore, we intended to examine whether the scale “behaves as expected concerning known groups” (i.e., known-group comparison approach) [36]. The method of known groups is a typical method for supporting construct validity and is given when a test can discriminate between a group of people known to have a specific characteristic and a group who do not have the characteristic. The instrument’s total score was compared based on gender and insurance coverage [37–40].

To investigate the factorial structure of the questionnaire, the EFA was used. The principal component analysis (PCA) and varimax rotation were performed to conduct EFA. Assuming the factors are not correlated with each other and are independent, rotation of the orthogonal method and the standard method of varimax was used.

To confirm the hypothesis, EFA, Kaiser-Meyer-Olkin (KMO), and Bartlett’s test were performed. The KMO test is used in EFA to determine how suited the data is and measure the sampling adequacy. Bartlett’s test of sphericity is used to assess an adequate amount of correlation (*p* < 0.05) between items. Scree plot was used to determine the number of factors. The parallel analysis and scree plot were also conducted to determine the accurate number of factors compared with eigenvalues, which tend to overestimate the number of factors [41]. All data processes were conducted and analyzed using SPSS software version 16.0.

#### Reliability assessment

The reliability assessment of the dimensions was measured using internal consistency with Cronbach’s alpha. According to Nunnally and Bernstein (1994), newly developed instruments with Cronbach’s alpha of more than 0.5 are acceptable, but the threshold for other cases is 0.7 [42].

#### Ethical considerations

The Ethics Committee of Kerman University of Medical Sciences approved this study (Approval ID: IR.KMU.REC.1397.040). After explaining the study’s objectives to the participants, written informed consent forms were obtained from them, and they were ensured about the confidentiality of their information. All participants were told to withdraw from the study at any time.

## Results

In this section, the results are reported according to the steps of the study.

### Stage one: literature review and qualitative inquiry

From the literature review and qualitative interviews analysis in the first stage, 30 and 48 items, respectively, were extracted. After removing duplicates and similar items, 57 items were obtained as items of the questionnaire’s initial version.

### Stage two: evaluation of the psychometric properties of the instrument

At this stage, the analysis of the instrument’s psychometric properties, including content validity, formal validity, construct validity, and reliability (internal consistency), were reported.

### Validity assessment

At this stage, after obtaining experts’ comments, CVR and CVI values were calculated to evaluate the content validity of the questionnaire, and 18 items achieved acceptable CVR and CVI values and were approved.

The instrument’s content validity results were as follows: of 57 items, the item content validity index (I-CVI) for 42 items was higher than the acceptable value of 0.78 (I-CVI = 1 for 24 items and I-CVI = 0.8 for 18 items). Item content validity ratio (I-CVR = 1) for 18 items was higher than the acceptable value of 0.99. The scale content validity ratio (S-CVR), and the scale content validity index (S-CVI) were also obtained. The S-CVR and S-CVI for the final 18 items were equal to 1.0 and higher than 0.78. Finally, 18 items obtained the acceptable value for both indicators and were confirmed.

In the stage of evaluation of formal validity, of 57 items, some items were underscored based on the experts’ opinion due to repetition, overlapping with other items, information gaps, and information asymmetry between patients or recipients of the health care service and health care system (health care provider) in response to the item. According to the suggestions by the experts about items’ wording and arrangement, changes were made.

We interviewed ten patients to evaluate the comprehensibility of the items of the questionnaire. According to their comments, some examples were added to some items to meet the fluency and better understanding.

### Demographic characteristics

The questionnaire was distributed to four hundred and forty samples. Four hundred out of 440 questionnaires (response rate = 90.9%) was received entirely and returned. Among patients referred to outpatient clinics, 184 (46%) were male, and 216 (54%) were female. The age of most participants was 30 to 60 years. Most of the patients (70.75%) were married. About 13% of them had an academic education. About 50% of the patients were employed, and the remaining were unemployed or homemakers. Most patients (92.25%) were residents of Kerman province. Only about one-fourth of the patients were living in the center of the province. More than 90% of the participants had basic insurance, and only 11% had supplementary health insurance. In terms of economic status, almost half of the patients (53.55%) had an income between 10 and 20 million Rials (IRR). All participants had Iranian nationality (See Table 1).

**Table 1:** Descriptive statistics of demographic variables of patients referred to outpatient clinics in Kerman (*n* = 400)

Variable	Frequency (%)
**Age**	
< 30 years	102 (25.50)
30–45 years	137 (34.25)
45–60 years	129 (32.25)
> 60 years	32 (8)
**Gender**	
Male	184 (46)
Female	216 (54)
**Marital status**	
Single	83 (20.75)
Married	283 (70.75)
Divorced	10 (2.50)
Widower/widow	24 (6)
**Education**	
Illiterate	75 (18.75)
Elementary	64 (16)
< diploma	123 (30.75)
Diploma	84 (21)
> diploma	54 (13.50)
**Occupational status**	
Unemployed	212 (53)
Employed	188 (47)
**Province of residence**	
Kerman	369 (92.25)
Sistan & Baluchestan	28 (7)
Fars	1 (0.25)
Hormozgan	2 (0.50)
**Residential area**	
Center of province (Capital)	103 (25.75)
County	117 (29.25)
Town	121 (30.25)
Village	59 (14.75)
**Basic health insurance**	
No	13 (3.25)
Yes	387 (96.75)
**Supplementary insurance**	
No	355 (88.97)
Yes	44 (11.03)
**Family income (Rials)**	
< 1 0 million	64 (16.24)
10–20 million	211 (53.55)
20–30 million	88 (22.34)
> 30 million	31 (7.87)

### Exploratory factor analysis

Kaiser-Meyer-Olkin (KMO) test for each item was higher than 0.5 with an overall value of 0.73 for the whole questionnaire, which is above 0.6, indicating that the sample size and factorability are met [43] for conducting EFA. The significance level of the Bartlett test Bartlett’s test of sphericity was less than 0.05. Therefore, it indicates an adequate amount of collinearity (*p* < 0.05) between items.

**Fig. 1:**
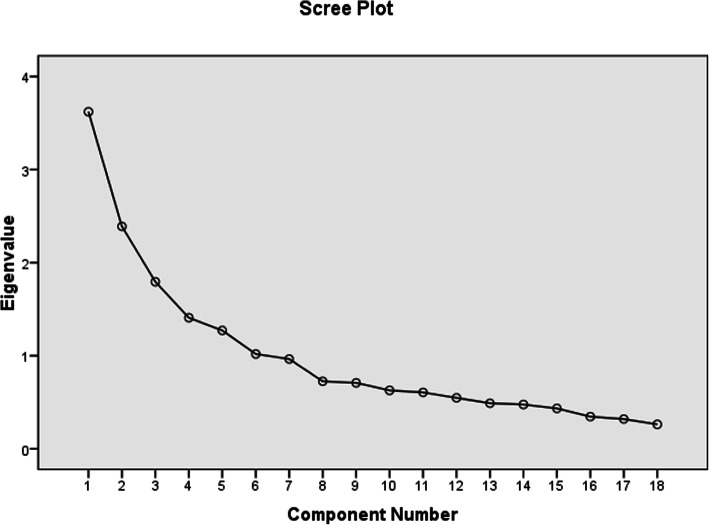
Scree plot test based on EFA for patients’ non-adherence with medical orders in outpatient clinic settings. Note. EFA = exploratory factor analysis

The scree plot with a four-factor structure with 18 items obtained after performing the PCA, considered the most suitable questionnaire structure (See Fig. 1). As shown in Table 2, the initial eigenvalues for all four factors are higher than one.

**Table 2:** Statistical indices of exploratory factor analysis using principal components analysis

Component	Initial eigenvalues	Rotation sums of squared loadings	
	Total	% of variance	Cumulative %
1	3.617	15.057	15.057
2	2.393	14.618	29.676
3	1.792	12.433	42.109
4	1.406	9.045	51.154

Approximately 51% of the total variance was explained by the four factors (factor 1 = 15.05%, factor 2 = 14.61%, factor 3 = 12.43%, and factor 4 = 9.04%). After rotation, items 3, 4, 7, and 8 were loaded with the first factor, items 10–15 were loaded with the second factor, items 1, 2, 5, 6, and 9 were loaded with the third factor, and items 16–18 were loaded with the fourth factor (Table 3). According to the items’ content in each factor, intrapersonal-psychological, provider-related, intrapersonal-cognitive, and socio-economic reasons seem to be appropriate as perceived reasons by patients for the factors.

**Table 3:** Rotated component matrix of each item using exploratory factor analysis with principal component analysis method

Item	Component			
	1	2	3	4
7. Hastiness and hasty judgment on the desirable treatment outcomes	.822			
4. Others’ advice (changing the current physician, non- adherence with therapeutic recommendations of the physician)	.693			
8. Fear of the consequences of the treatment and diagnostic modalities (e.g., endoscopy, colonoscopy, and positive test results)	.677			
3. My unpleasant experience of my disease treatment in the past (I did not receive desirable outcomes after treatment)	.564			
12. Inadequate time allocated by physician to visit patient		.773		
10. Inappropriate behavior of physician (lack of a good eye contact, irascibility, etc.)		.705		
15. Prescription of unnecessary diagnostic and therapeutic measures (medicines, tests, etc.)		.693		
11. Inadequate physician expertise		.546		
13. Lack of access to telephone counseling with physician		.465		
14. The adverse effects of prescriptions (side effects of medicines and treatments such as the use of corticosteroid and radiology)		.439		
6. Disease is a fate and destiny and efforts to treat is useless.			.746	
1. My misunderstanding of the physician therapeutic recommendations (drug dosage, time and frequency of drug use and etc.)			.665	
9. Considering the treatment long duration			.629	
2. Lack of enough knowledge about my disease, diagnosis, and treatment	.301		.603	
5. The incompatibility of the treatment method with my preferences (unwilling to use ampoule or bad tasting medicine)			.330	−.686
16. Shortage of medicines and facilities	.334			.672
17. High cost of diagnostic and therapeutic procedures (visit, medicines, tests)				.550
18. Ongoing preoccupations of life			.383	.550

### Construct validity

Our findings for convergence among the adherence score and its relationship with respondents/patients’ demographic variables showed a significant relationship between non-adherence with gender and insurance coverage (*p* < 0.05). Non-adherence score was higher in men in comparison with the Females. Furthermore, patients who lack basic health insurance coverage have a higher score of non-adherence than insured patients (*p* < 0.05). These results are consistent with the literature and results of other existing standard instruments that indicate a convergence between our instrument and other existing instruments.

### Reliability assessment

Reliability assessment of the questionnaire (Average Cronbach’s alpha = 0.70) was confirmed. The Cronbach’s alpha coefficient for intrapersonal-psychological, intrapersonal-cognitive, provider-related, and socio-economic reasons was 0.70, 0.66, 0.73, and 0.71, respectively (Table 4). Cronbach’s alpha coefficient for intrapersonal-psychological reasons was less than the acceptable level of 0.7. This scale measures different characteristics to explain why the Cronbach’s alpha coefficient for this dimension was less than the acceptable level.

**Table 4:** Results of the reliability assessment using Cronbach’s alpha

Dimension	Cronbach’s alpha
Intrapersonal-psychological	0.70
Provider-related	0.73
Intrapersonal-cognitive	0.66
Socio-economic	0.71
Average	0.70

The medium value of Cronbach’s alpha coefficient can be due to this finding that about 50% of the participants had identical responses. Our EFA showed four dimensions of the scale, which means four different characteristics were on this scale. This point could explain why the coefficient alpha was not higher than 0.7 as an acceptable level. Moreover, if the number of test items of each scale is too small, the scale’s reliability would be underestimated [44]. The low variability in the scale scores especially the intrapersonal-cognitive scale is another reason for the Cronbach alpha lower than 0.7, which means that about half of the subjects in this sample had similar values. If variability between scale scores were greater, which would arise in a population with varying levels of adherence, internal consistency would be strengthened [45]. In 3 items related to intrapersonal-psychological reasons, more than half of the participants selected the number of 0 or 1, which decreased Cronbach’s alpha coefficient. The internal consistency reliability can be improved by increasing the number of responses [45]. However, the average Cronbach’s alpha coefficient of the questionnaire 0.70 and is considered acceptable. The final version of the developed questionnaire was presented in the Supplementary file 1.

## Discussion

This study was conducted to design and evaluate the psychometric properties and factor structure of a questionnaire to determine the perceived reasons for patients’ non-adherence with therapeutic recommendations in outpatient clinics. The EFA findings explain about 51% of the variance, indicating four dimensions of intrapersonal-psychological, intrapersonal-cognitive, provider-related, and socio-economic reasons.

Intrapersonal-psychological reasons include hastiness and hasty judgment, others’ advice, anxiety, fear and anxiety for the consequences of the diagnosis and treatment, and experiences of the individuals. The intrapersonal-cognitive reasons include individual perceptions, individual attitudes toward disease, health literacy, patient preferences, and values. The provider-related reasons can include factors such as inappropriate physician behavior, adverse effects of prescriptions, and unnecessary diagnostic and the therapeutic measures. The socio-economic reasons include the costs, problems, and concerns of life-related to social issues, family support, and access to facilities and medicines.

A study conducted in Ghana concluded that knowledge and experience act as influential factors for non-adherence [46]. Moreover, other studies showed that the relationship between physician and patient [47], patient involvement in the treatment process [48, 49], social attention, and support such as family support in helping to maintain continuous care and treatment [50, 51] affect decision making and determination of reasons for non-adherence. A systematic review and meta-analysis in Ethiopia also identified fear and waiting time as determinants of non-adherence [52]. Studies in Tanzania [53] and Australia [54] have also reported economic factors as reasons for non-adherence.

Instruments developed in previous studies show that these studies evaluated the reasons for non-adherence to a specific disease. In a study by Chizzola et al. (1996), adherence with therapeutic recommendations in patients with cardiovascular disease in outpatient clinics was investigated using an instrument measuring only socio-economic factors and patients’ knowledge of medications [55]. In a 14-item questionnaire designed by Jank et al. (2009), individuals’ attitudes towards medicines, medical care expenses, and daily life obstacles were evaluated [56]. Many items evaluated in the study are consistent with two dimensions of intrapersonal-psychological and intrapersonal-cognitive of the questionnaire proposed in our study, but other specific reasons have not been consistent with our instrument.

Muller et al. (2015) conducted a study to design an instrument for identifying medication non-adherence factors. Three categories of barriers and reasons were identified in this study. The first group refers to intentional non-adherence, indicating that patients consciously decide to deviate from the treatment plan due to their attitudes or negative beliefs. The second category was unintentional non-adherence, like forgetfulness, depression, or lack of knowledge about the importance of adherence. The third category was factors related to medicines and the healthcare system, affecting non-adherence with medication [57]. Some intentional or unintentional obstacles are consistent with the intrapersonal-psychological and intrapersonal-cognitive dimensions. Furthermore, the obstacles related to medicines and the healthcare system are limitedly consistent with provider-related reasons in the present study. However, there is no item for identifying the socioeconomic reasons for non-adherence.

Most instruments measuring non-adherence with therapeutic recommendations are restricted to specific patient groups or do not cover all the reasons for non-adherence. Studies have evaluated non-adherence in patients with schizophrenia [19], hypertension [20], HIV [21], and tuberculosis [58], but the instrument provided in this study can be used for all patients in outpatient clinics.

Weinman et al. (2018) carried out a study to validate a valid instrument for non-adherence. This measurement instrument was limited to the reasons for the intentional non-adherence. For validity and evaluation of the instrument’s factorial structure, patients were selected from three clinical groups, including hypertension, oncology, and gout [24]. However, the present study questionnaire encompasses intentional and unintentional non-adherence with therapeutic recommendations prescribed by physicians. This point may be usefully applied to measure the non-adherence with medical recommendations for all diseases and at all outpatient settings in a general way.

Morisky et al. (2008) Evaluated the validity of an instrument of non-adherence in outpatient settings. This instrument is only applicable to medication non-adherence and in patients with hypertension. Items for this instrument assessed various psychosocial reasons for non-adherence and social support, satisfaction with care, and complexity of the medical regimen [59].

The questionnaire provided in this study measures all areas of treatment (medicine, visit, diagnostic services, laboratory services, non-medication recommendations such as diet and rehabilitation services and physiotherapy), while most of the previous instruments significantly evaluated the area of medicine [60, 61]. Morisky et al. questionnaire was designed to evaluate medication adherence [62]. Ghada Asaad et al. (2015) reviewed the validity of an instrument measuring dietary adherence in patients with type 2 diabetes [63].

As expected from relevant studies [37–40], the results of “known group comparisons” showed that females and patients with insurance had better adherence than male patients and those who lacked insurance.

### Study limitations and strengths

One of the limitations of this study is that the questionnaire is limited to outpatient clinics and is designed to assess the reasons for non-adherence with medical orders in these settings and cannot be used in other health service providers and clinical environments such as hospitals. Also, according to the different contexts in countries, results may not be generalized to other countries. Another limitation is that one dimension’s reliability is less than the acceptable level, and further studies are needed to modify the items. Another limitation of this study is excluding some reasons, such as waiting time for visit, severity, and duration of diseases, lack of desired outcomes/meeting patients’ expectations, payment system, undesirable medication dosage prescribed by the physician, the cost-effectiveness of the visit and its effect on the frequent referral to different physicians. It should be noted that the experts excluded these factors as some of them were considered unnecessary. Some other reasons were excluded because the experts believed that patients have no knowledge and awareness about the health system’s features.

Because the questionnaire was designed based on the patients’ perspective, there are many essential limitations on conducting surveys on this group that should be considered, including the short form of the questionnaire, questionnaire completion time, and respondents’ patience in crowded healthcare centers increase its applicability. Long-form questionnaires are one reason for biased responses and can produce low-quality data [64, 65]. Also, it makes respondents bored and results in incorrect responses or less response rate [66]. Therefore, it can reduce the explanatory variance to a lower extent, which needs further research to design a longer form of a questionnaire with more components in other settings that can be more comprehensive. Last but not least, another limitation is, we did use no theoretical base of behavior change models, considering this issue that there are many predictors of non-adherence [67]. It is suggested that later research be focused on this issue.

Some reasons for non-adherence with medical orders may differ for different diseases and conditions [20, 58, 63]. However, the present study’s key strength is that the questionnaire identifies general reasons for non-adherence with therapeutic recommendations from patients’ perspectives in outpatient clinics; since there was no questionnaire to identify patients’ general behavior regardless of the type of disease and prescription in the outpatient clinics.

The present questionnaire can be used in outpatient clinics, and it is a recommendation for future studies, a questionnaire developing for non-adherence in inpatient settings. The questionnaire was also designed based on the patients’ perspectives, which recommendations for future studies to a questionnaire developing for determining the reasons for non-adherence from the providers’ perspectives.

## Conclusion

According to the results, the questionnaire with four dimensions derived from exploratory factor analysis includes all dimensions of the perceived reasons for patients’ non-adherence with therapeutic recommendations. It is a valid and reliable instrument for identifying the perceived reasons for non-adherence with medical orders. Most of the similar instruments have measured medication non-adherence or have been limited to certain diseases. However, patients’ treatment is not solely based on medication non-adherence. This questionnaire is not limited to a specific area of non-adherence. It includes medical orders such as medicine-related recommendations, diagnostic services, and laboratory non-medication recommendations such as diet, physiotherapy, and rehabilitation services. This questionnaire can help policymakers identify the general reasons for non-adherence from patients’ perspectives in providing outpatient services, and as a result, to adopt tailored and effective policies and interventions to reduce non-adherence with adequate health care utilization and continuity of care. The questionnaire has good psychometric properties and can be used to investigate the reasons for non-adherence with therapeutic recommendations in outpatient clinics.

## Supplementary Information

**Additional file 1.** Final version of the developed questionnaire.

## Data Availability

The datasets analyzed during the current study are available from the corresponding author on reasonable request.

## Abbreviations

EFA: Exploratory Factor Analysis
PCA: Principal Component Analysis
