# Supplementary material for: Development and validation of a questionnaire to determine medical orders non-adherence: a sequential exploratory mixed-method study
Source: BMC Health Serv Res. 2021 Feb 12;21:136. doi: 10.1186/s12913-021-06147-3 (PMC7881677; doi:10.1186/s12913-021-06147-3)
Supplement: Supplementary file 1 — Additional file 1. Final version of the developed questionnaire. [file 12913_2021_6147_MOESM1_ESM.docx]

**Supplementary file 1:** Final version of the developed questionnaire

**Questionnaire to Determine the Perceived Reasons of Patients' Non-** **adherence with Therapeutic Recommendations of Physicians in the Outpatient Clinics (Based on the Patients' Perspectives)**

Have you had any experience of non-adherence with medical orders of a physician during the last four weeks?

□ Yes

□ No

If yes, please check mark the importance of each of the following items that describes your non- adherence with therapeutic recommendations of physicians.

| **Items** | **Scale** | | | | |
| --- | --- | --- | --- | --- | --- |
|  | **Very Unimportant (0)** | **Unimportant (1)** | **Slightly Important (2)** | **Important (3)** | **Very Important (4)** |
| 1. My misunderstanding of the physician therapeutic recommendations (drug dosage, time and frequency of drug use and etc.) |  |  |  |  |  |
| 2. Lack of enough knowledge about my disease, diagnosis, and treatment |  |  |  |  |  |
| 3. My unpleasant experience of my disease treatment in the past (I did not receive desirable outcomes after treatment) |  |  |  |  |  |
| 4. Others' advice (changing the current physician, non- adherence with therapeutic recommendations of the physician) |  |  |  |  |  |
| 5. The incompatibility of the treatment method with my preferences (unwilling to use ampoule or bad tasting medicine) |  |  |  |  |  |
| 6. Disease is a fate and destiny and efforts to treat is useless. |  |  |  |  |  |
| 7. Hastiness and hasty judgment on the desirable treatment outcomes |  |  |  |  |  |
| 8. Fear of the consequences of the treatment and diagnostic modalities (e.g., endoscopy, colonoscopy, and positive test results) |  |  |  |  |  |
| 9. Considering the treatment long duration |  |  |  |  |  |
| 10. Inappropriate behavior of physician (lack of a good eye contact, irascibility, etc.) |  |  |  |  |  |
| 11. Inadequate physician expertise |  |  |  |  |  |
| 12. Inadequate time allocated by physician to visit patient |  |  |  |  |  |
| 13. Lack of access to telephone counseling with physician |  |  |  |  |  |
| 14. The adverse effects of prescriptions (side effects of medicines and treatments such as the use of corticosteroid and radiology) |  |  |  |  |  |
| 15. Prescription of unnecessary diagnostic and therapeutic measures (medicines, tests, etc.) |  |  |  |  |  |
| 16. Shortage of medicines and facilities |  |  |  |  |  |
| 17. High cost of diagnostic and therapeutic procedures (visit, medicines, tests) |  |  |  |  |  |
| 18. Ongoing preoccupations of life |  |  |  |  |  |
